# Etiology of Hepatocellular Carcinoma: Special Focus on Fatty Liver Disease

**DOI:** 10.3389/fonc.2020.601710

**Published:** 2020-11-30

**Authors:** Diwakar Suresh, Akshatha N. Srinivas, Divya P. Kumar

**Affiliations:** Department of Biochemistry, Center of Excellence in Molecular Biology and Regenerative Medicine (CEMR), Jagadguru Sri Shivarathreeshwara (JSS) Medical College, JSS Academy of Higher Education and Research, Mysuru, India

**Keywords:** hepatocellular carcinoma, alcoholic fatty liver disease, non-alcoholic fatty liver disease, etiology, metabolic syndrome, hepatitis viruses

## Abstract

Hepatocellular Carcinoma (HCC) is a highly aggressive cancer with mortality running parallel to its incidence and has limited therapeutic options. Chronic liver inflammation and injury contribute significantly to the development and progression of HCC. Several factors such as gender, age, ethnicity, and demographic regions increase the HCC incidence rates and the major risk factors are chronic infection with hepatitis B virus (HBV) or hepatitis C virus (HCV), carcinogens (food contaminants, tobacco smoking, and environmental toxins), and inherited diseases. In recent years evidence highlights the association of metabolic syndrome (diabetes and obesity), excessive alcohol consumption (alcoholic fatty liver disease), and high-calorie intake (nonalcoholic fatty liver disease) to be the prime causes for HCC in countries with a westernized sedentary lifestyle. HCC predominantly occurs in the setting of chronic liver disease and cirrhosis (80%), however, 20% of the cases have been known in patients with non-cirrhotic liver. It is widely believed that there exist possible interactions between different etiological agents leading to the involvement of diverse mechanisms in the pathogenesis of HCC. Understanding the molecular mechanisms of HCC development and progression is imperative in developing effective targeted therapies to combat this deadly disease. Noteworthy, a detailed understanding of the risk factors is also critical to improve the screening, early detection, prevention, and management of HCC. Thus, this review recapitulates the etiology of HCC focusing especially on the nonalcoholic fatty liver disease (NAFLD)- and alcoholic fatty liver disease (AFLD)-associated HCC.

## Introduction

Hepatocellular Carcinoma (HCC) is a serious public health issue and the fourth leading cause of cancer mortality worldwide (1, 2). HCC accounts for about 80% of the primary liver cancer while the other types include cholangiocarcinoma (10–20%) and angiosarcoma (1%) (3). There is a striking variation in HCC incidence rates across geographic regions and at the global level, each year over 800,000 people are diagnosed with liver cancer (4, 5). HCC cases are highest in Eastern Asia and sub-Saharan Africa, followed by intermediate rates in Southern and Western European countries, North and Central America, and the lowest incidence rates are observed in and Northern Europe and South Central Asia (6, 7). HCC predominantly affects men more than women (two to four times higher in men) with its highest incidence in the age group of 45–65 years (8, 9). According to Globocan 2018, HCC is the fifth most common cancer in men and the ninth most commonly occurring cancer in women (10). The overall ratio of mortality to incidence is 0.95 and reflects the poor prognosis of HCC (11).

**Figure 1:**
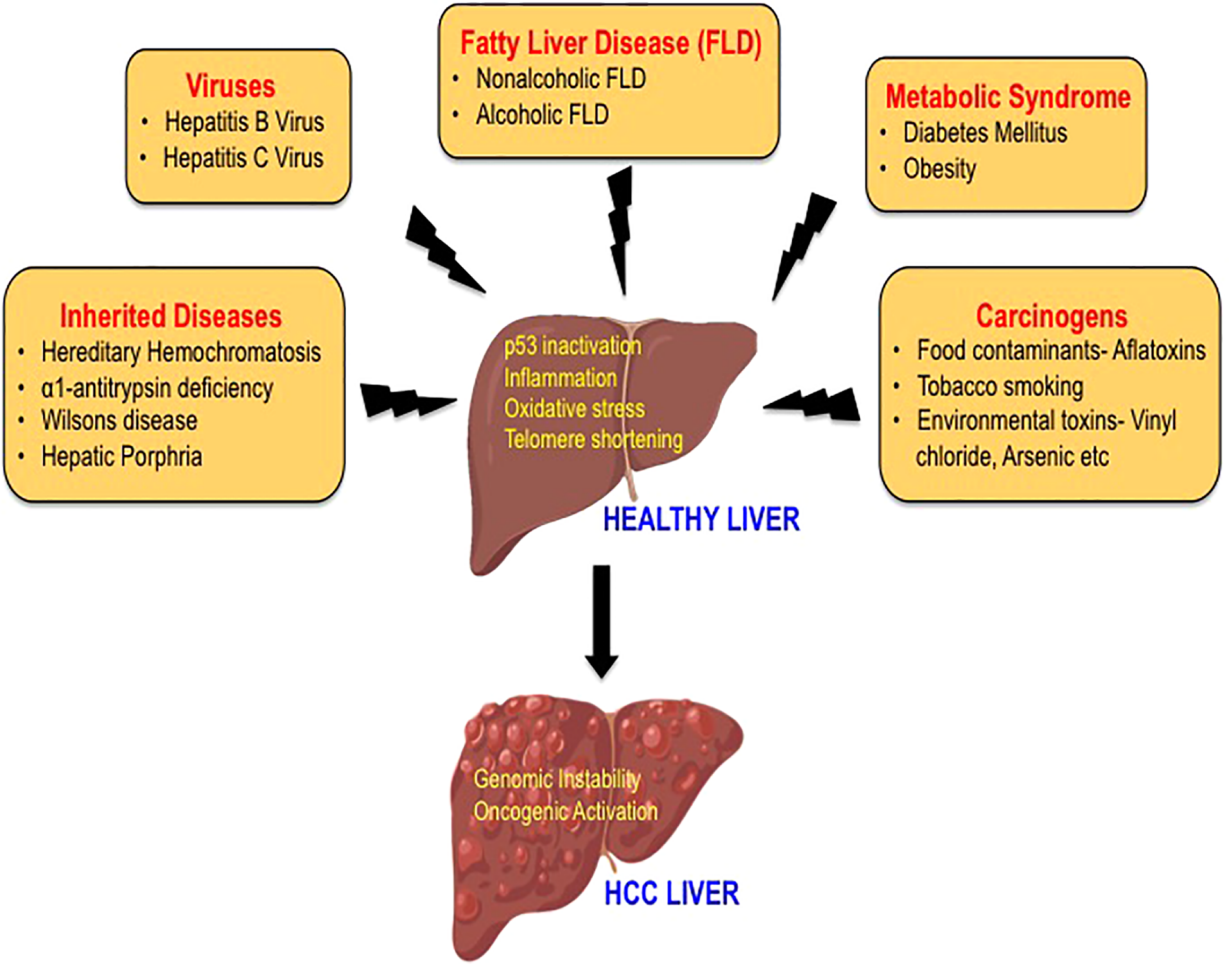
The etiology of hepatocellular carcinoma. A variety of risk factors have been associated with the development of HCC, including hepatitis viruses, carcinogens, heredity diseases, metabolic syndrome, and fatty liver disease. The mechanisms by which these etiological factors may induce hepatocarcinogenesis mainly include p53 inactivation, inflammation, oxidative stress, and telomere shortening leading to genomic instability and activation of multiple oncogenic signaling pathways.

HCC is an extremely complex condition and there are multiple factors involved in the etiology of HCC. The major risk factors for HCC include hepatitis B virus (HBV) and hepatitis C virus (HCV), diabetes, obesity, alcoholic fatty liver disease (AFLD), and non-alcoholic fatty liver disease (NAFLD). Additional risk factors that are also known to increase the incidence of HCC are tobacco smoking, food contaminants such as aflatoxins, familial or genetic factors, and various environmental toxins that act as carcinogens (12–14) (**Figure 1**). The development of HCC is initiated by hepatic injury involving inflammation leading to necrosis of hepatocytes and regeneration. This chronic liver disease sequentially transitions to fibrosis, cirrhosis, and hepatocellular carcinoma (15, 16). HCC that often occurs in the setting of chronic liver disease and cirrhosis is diagnosed late in its course and liver transplantation is the best option for patients at this stage (12, 17). Multiple treatment options are available to treat HCC including surgical resection, local ablation with radiofrequency, transcatheter arterial chemoembolization (TACE), radioembolization, and systemic targeted agents like sorafenib depending on the tumor extent or underlying liver dysfunction (12, 14, 18). Furthermore, the viable treatment options offered to the patients also depend on the causative agent of HCC as they define the disease course and patient characteristics. However, with the improved treatment for HCC, the demographic landscape has changed (6, 19). In this mini-review, we aim to describe the traditional risk factors in brief and highlight on fatty liver disease, which is the emerging etiological risk factor contributing to the increasing incidences of HCC.

## Virus and HCC

The chronic infection by hepatitis B virus (HBV) and hepatitis C virus (HCV) are the traditional risk factors that are associated with HCC for 33,600 years and 1,000 years, respectively (20, 21). The virus-associated mechanisms driving hepatocarcinogenesis are complex and cause liver cirrhosis, which progresses to HCC in about 80–90% of the cases (15, 22).

HBV is partially a double-stranded circular DNA virus, which belongs to the genus *Avihepadnavirus* of the Hepadnaviridae family. HBV infection accounts for 75–80% of virus-associated HCC and infects over 240 million people around the world (23). The incorporation of the genetic material of this virus into the human genome causes p53 inactivation, inflammation, or oxidative stress, which causes hepatocarcinogenesis (24, 25). HBV-induced HCC can be both cirrhotic and non-cirrhotic and involves an array of processes such as proliferation and loss of growth control (caused by p53 inactivation), sustained cycles of necrosis and regeneration (resultant of inflammation), and activation of various oncogenic pathways such PI3K/Akt/STAT3 pathway and Wnt/β-catenin (induction of oxidative stress), all of which leads to genomic instability (26, 27).

Contrary to HBV, the Hepatitis C virus (HCV) is a non-integrating, single-stranded RNA virus belonging to the genus *Hepacivirus* of the Flaviviridae family. HCV infects over 57 million people worldwide and accounts for 10–20% of virus-associated HCC (28, 29). Unlike HBV infection, there is no integration of genetic material into the host’s genome by the HCV virus. It is the HCV proteins (structural and non-structural proteins) that play a critical role in the development of HCC (30). HCV-induced hepatocarcinogenesis is highly complex involving the activation of multiple cellular pathways and gets initiated by the establishment of HCV infection leading to chronic hepatic inflammation, which further progresses to liver cirrhosis and HCC development (31). HCV proteins either directly or indirectly modulate a wide range of host cellular activities, including transcriptional regulation, cytokine modulation, hepatocyte growth regulation, and lipid metabolism that lead to chronic liver injury. In addition to inducing oxidative stress and endoplasmic reticulum (ER) stress, HCV proteins are also known to cause epigenetic alterations by modulating micro RNA (miRNA) and long noncoding RNA (lncRNA) in the host cells (32). Thus, HCV shows a high propensity (60–80%) to induce chronic infection and promotes liver cirrhosis 10–20 fold higher than HBV. The angiogenic and metastatic pathways activated by HCV further promote hepatocytes’ malignant transformation and accelerate HCC development (33). Hepatitis D virus (HDV) and human immunodeficiency virus (HIV) are also considered as modulators of HCC (14).

## Carcinogens and HCC

In addition to hepatitis viruses, chemical carcinogens also play important roles in the etiology of HCC (34). Exposure to carcinogens including aflatoxins, tobacco smoking, vinyl chloride, arsenic, and various other chemicals act either independently or in combination with viruses to cause DNA damage, induce liver cirrhosis, and contribute to HCC (35).

Aflatoxin is a potent liver carcinogen produced by the Aspergillus fungus, which is found to contaminate foodstuffs such as peanuts, corn, soya beans stored in damp conditions. This mycotoxin induces mutation in the p53 tumor suppressor gene and causes uninhibited growth of liver cells leading to the development of HCC (36, 37). It is reported that the chemicals in tobacco smoke (4-aminobiphenyl and polycyclic aromatic hydrocarbons), areca nut (nitrosamines), and betel leaves (safrole) cause hepatotoxicity (13, 35). Besides, studies have demonstrated that the human exposure to groundwater contaminants (chemicals such as cadmium, lead, nickel, arsenic), organic solvents (toluene, dioxin, xylene), and chemicals such as vinyl chloride and dichlorodiphenyltrichloroethane (DDT) have shown to increase the risk of HCC as they exert hepatocarcinogenic effect *via* induction of oxidative stress and telomere shortening (34, 38).

## Inherited Diseases and HCC

Certain metabolic disorders such as hereditary hemochromatosis, α1-antitrypsin deficiency, Wilson’s disease, and hepatic porphyria are associated with high risk for the development of HCC. These hereditary diseases are known to promote hepatocarcinogenesis as a result of increased inflammation and hepatocellular damage (39–41).

## Metabolic Syndrome and HCC

Diabetes mellitus, a component of the metabolic syndrome has been shown to attribute about 7% of the HCC cases worldwide (5, 42). Meta-analyses have shown that diabetes is associated with HCC independent of viral hepatitis in which diabetic patients show 2-3 fold greater risks in developing HCC compared with non-diabetic controls (43). The pathophysiological conditions such as hyperglycemia, hyperinsulinemia, insulin resistance, and activation of insulin-like growth factor signaling pathways provide a strong association for diabetes to be the risk factor in the pathogenesis of HCC (5, 44). Obesity, a pathological state characterized by insulin resistance, hyperinsulinemia, and inflammation is also closely associated with HCC (45). It is demonstrated that increased reactive oxygen species, dysregulated adipokines, and adipose tissue remodeling, alteration of gut microbiota, and dysregulated microRNA increases the relative risk of HCC in obese patients (46–48). Accordingly, obesity is one of the common causes of NAFLD, which is also an underlying risk factor of HCC (46).

## Fatty Liver Disease and HCC

**Figure 2:**
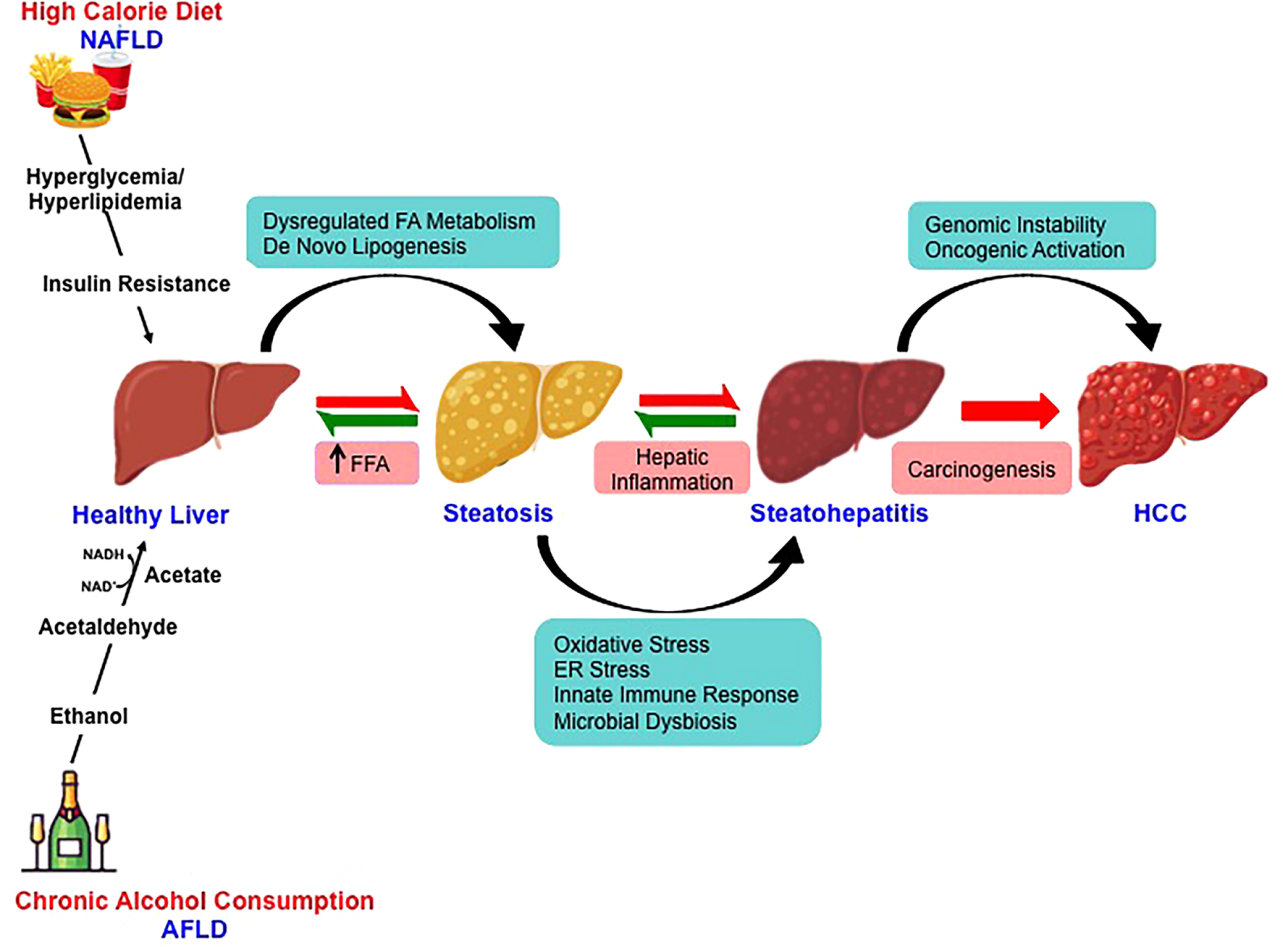
Molecular mechanisms involved in nonalcoholic- and alcoholic-associated HCC. High-calorie diet and excessive alcohol consumption is the major risk factor for the development of NAFLD and AFLD respectively. Despite the divergent pathogenic origin, the pathological spectra of liver injury in promoting HCC development in NAFLD and AFLD share common molecular pathways.

Over the last decade, fatty liver disease is emerging as the leading etiologies for chronic liver disease progressing to HCC (49). The changing scenario is attributed to improved antiviral therapy for virus-related HCC (50). With the growing inclination towards western dietary pattern, sociocultural changes and the lifestyle with limited or no physical activity has sharply increased the incidence rates of NAFLD- and AFLD-associated HCC across the continents (51, 52). The pathological spectra of liver injury in promoting HCC development are similar in these two fatty liver diseases despite having divergent pathogenic origin with yet some key distinct features (**Figure 2**). Furthermore, a high-calorie diet and ethanol act synergistically at multiple levels potentiating hepatocarcinogenesis (53).

### Non-Alcoholic Fatty Liver Disease (NAFLD)-Associated HCC

NAFLD is characterized by excessive hepatic lipid accumulation (steatosis), which further transitions to steatohepatitis upon the inflammatory insult, to cirrhosis and HCC (54, 55). It’s a pathophysiological condition that is not associated with excess alcohol consumption or other secondary causes such as viral infection and heredity liver diseases (56). NAFLD is classically associated with metabolic disorders such as obesity, hypertension, dyslipidemia, insulin resistance, and type 2 diabetes (57, 58).

A meta-analysis by Younossi et al. (86 studies from 22 countries carried out between 1989 and 2015) reported that the worldwide prevalence of NAFLD is 25.24% (59). The prevalence of NAFLD varies across the continent with the highest in the Middle East (31.79%) followed by South America (30.45%), Asia (27.37%), North America (24.13%), Europe (23.71%), and Africa (13.48%) (51, 60). Studies also indicate that NAFLD is more common in men (42% for white males *vs.* 24% for white females) and the prevalence of NAFLD increases with age (61, 62). However, as obesity increases in children and adolescents, there is an increasing prevalence of NAFLD and NAFLD-associated HCC compared to adults (63, 64). While studies have shown that NAFLD accounts for about 13% of HCC cases, Wong et al., have reported that NAFLD is the fastest-growing etiology, which is indicative of liver transplantation in HCC patients (65). Studies from long term follow up of non-alcoholic fatty liver patients have shown the prevalence of HCC to be 0.5 and 2.8% in NAFLD and NASH respectively (66, 67). It is interesting to note that 80% of HCC patients have cirrhosis (68). However, HCC is also reported in non-cirrhotic NASH (69). Thus, with the rise in the incidence of NAFLD-associated HCC in recent years, the contribution of NAFLD is underscored among the risk factors that induce HCC (70).

Emerging evidence has established multiple risk factors for NAFLD-associated HCC including obesity, diabetes, iron deposition, genetic and epigenetic factors, microRNA, and gut microbiota (49, 71). In the modern era with a sedentary lifestyle and unhealthy dietary habits, obesity is rapidly increasing and has been established as a risk factor for HCC (56). It is been reported to increase the risk by 1.5–4 times either by contributing to the development of NAFLD or by directly exerting carcinogenic effect leading to HCC (72). Albeit most patients with NAFLD are obese in the western countries, lean NAFLD has also been reported from Asian countries (73). Furthermore, large population-based cohort studies have found that diabetes mellitus is associated with 1.8–4 fold increased risk of HCC (74). Along the same line, a study by Turati et al. reported that the combined effect of diabetes and obesity among the metabolic syndrome was positively associated with HCC risk (75). Excessive iron deposit in the liver is thought to be a risk factor for NAFLD-HCC (76). Indeed, experimental studies by Paola et al., demonstrated that hepatic iron overload might be associated with HCC development in NASH patients (77). Additionally, genetic factors are known to increase the risk of HCC in NAFLD such as the PNPLA3 I148M variant and rs58542926 (E167K) variant in TM6SF2 (78, 79). Studies carried out in mouse models of NAFLD and also in patients with NAFLD or HCC have identified epigenetic-mediated gene regulation involved in the development and progression of the disease (80, 81). Among the various risk factors, the gut microbiota has emerged as an important contributor to NAFLD-associated HCC (82).

The mechanism of NAFLD-associated HCC progression is complex. Hepatic lipid accumulation as a result of high-calorie intake (high carbohydrate and high dietary fat) and low physical activity in the absence of excessive alcohol consumption is a major contributor to the onset of NAFLD development (56). Steatosis progresses to necroinflammation leading to hepatocarcinogenesis as a consequence of multiple parallel acting conditions such as insulin resistance, hyperinsulinemia, dyslipidemia, adipose tissue remodeling, oxidative/endoplasmic reticulum (ER) stress, altered immune system, genetic alterations, and dysbiosis in the gut microbiome. These modifications in association with genetic factors and epigenetic changes activate oncogenic signaling and promote HCC development (83). Insulin resistance leads to increased release of free fatty acids (FFA) and release of various inflammatory cytokines including tumor necrosis factor- α (TNF-α), interleukin 6 (IL-6), leptin, and resistin. This is also accompanied by decreased amounts of adiponectin (84). Insulin resistance along with hyperinsulinemia up-regulates insulin and insulin-like growth factor (IGF-1), a growth stimulator aiding hepatocyte proliferation and apoptosis inhibition (85, 86).

Furthermore, hepatic lipotoxicity due to insulin resistance leads to imbalanced energy metabolism. Elevated FFAs β-oxidation induces oxidative stress through the release of reactive oxygen species (ROS) eventually leading to mitochondrial dysfunction accompanied by ER stress (87, 88). There exists a potent cross talk between oxidative/endoplasmic reticulum (ER) stress, and apoptotic pathways along with inflammatory cytokines, innate and adaptive immune responses that significantly contribute to NASH progression to HCC (83). Further, the oxidative stress promotes tumorigenesis by activation of c-Jun amino-terminal kinase 1 (JNK1), a mitogen-activated protein kinase, and by suppressing the action of p53 tumor suppressor gene and nuclear respiratory factor 1 (Nrf1) (89). Interestingly, studies have confirmed the potential role of immune cells such as CD8^+^, CD4^+^ T lymphocytes, and Kupffer cells in NASH progression with altered intestinal gut microbiome being one of the contributors (90, 91). Thus, the molecular connection between regulations of hepatocyte cell cycle and energy balance is the key driving force of NAFLD-associated HCC.

Unfortunately, there is yet no FDA-approved drug for the effective treatment of NAFLD and NAFLD-HCC. A better understanding of the cellular and molecular mechanisms will open up treatment options for HCC subjects with NAFLD etiology. Dietary and lifestyle modifications being the mainstay of disease management need to be tailored to meet individual patients’ needs. Furthermore, knowing the co-morbidities of NAFLD-HCC will aid in designing effective treatment strategies that can be employed in clinical practice.

### Alcoholic Fatty Liver Disease (AFLD)-Associated HCC

As the name suggests, AFLD is attributed to excessive alcohol consumption that causes hepatic injury by the build-up of fats, inflammation, and scarring leading to HCC, which could be fatal (92). Globally, the prevalence of AFLD is increasing and has become a significant contributor to the liver disease burden accounting for 30% of HCC related deaths (93). The “safe” levels of drinking as defined in the dietary guidelines in the United States is two drinks for men and one drink for women per day as one alcoholic drink (12 ounces of beer, 5 ounces of wine, or 1 ounce of hard liquor) accounts for about 14 g of alcohol (defined as standard drink by WHO) (53). By contrast, excessive alcohol consumption (more than 14 drinks/week and 7 drinks/week for men and women respectively) is considered to cause AFLD (51). The threshold level of alcohol intake causing hepatotoxic effect varies and it depends on a variety of factors such as gender, ethnicity, and genetics (94).

A large population-based prospective study conducted by Becker et al., for 12 years have provided evidence that females are more susceptible to the toxic effects of alcohol than male for any given level of alcohol intake (95). The possible mechanisms include lower gastric alcohol dehydrogenase (ADH) activity in females and estrogen levels that activate Kupffer cells due to increased gut permeability and portal endotoxin levels leading to alcohol-induced liver injury (96, 97). Furthermore, studies have demonstrated that in the United States, compared to Whites, Blacks, and Hispanics drinkers have a two-fold increase in liver enzymes (98). Since there is no significant difference among other ethnic groups, factors such as polymorphism of genes associated with alcohol metabolism (*ADH*, *CYP2E1*) and antioxidant enzymes and genes coding for cytokines are also investigated in association with alcoholic liver disease (99). However, it remains critical to consider factors such as amount and type of alcohol consumption and socioeconomic status with the development of AFLD.

As per the global status report on alcohol and health, 2018, there are 2.3 billion active drinkers worldwide (100). In America, Europe, and Western Pacific more than half of the population account for active alcoholics. Though the percentage of drinkers has decreased in Africa and America, there is an increase observed in the Western Pacific region and has remained stable in the regions of Southeast Asia (101). Alcohol is one of the commonest causes of chronic liver disease with nearly 75 million diagnosed for the risk of AFLD and contributes to 50% of mortality related to cirrhosis (102). According to the global health report on alcohol and health, 2018 by World Health Organization (WHO), the alcohol-attributable deaths (AAD) from liver cirrhosis varies across the countries. The top five in the list includes India (Safe limits: ≤16 g/day for men and ≤8 g/day for women, Comparison of international alcohol drinking guidelines, 2019), China (Safe limits: ≤25 g/day for men and ≤15 g/day for women, Chinese Dietary Guidelines, 2016), Nigeria (Safe limits: no written national policy, WHO, 2018), United States (Safe limits: ≤24 g/day for men and ≤14 g/day for women, Dietary Guidelines for Americans 2015–2020), and Russia (Safe limits: ≤30 g/day for men and ≤20 g/day for women, Prevention of alcohol and drug use, National Medicine Research Center for Therapy and Preventive Medicine). It is also reported that liver cancer (22.5%) is the largest contributor to the burden of alcohol-attributable cancer DALY (disability-adjusted life year), followed by colorectal (20.6%) and esophageal (18.5%) cancers (100). The global HCC BRIDGE study by Park et al. reported that AFLD contributes to HCC development to a large portion in Europe (37%) and North America (21%) compared to East Asia (4–13%) (103). Furthermore, progression to cirrhosis and mortality is higher in patients with AFLD (36%) compared to NAFLD (7%) (104) and studies have reported that AFLD accounts for 10.3% of HCC in liver transplantation candidates (105). It is noteworthy that there is a synergy between excessive alcohol consumption with other risk factors including diabetes mellitus and viral hepatitis (106).

Despite the differences in the epidemiological and clinical characteristics, AFLD-associated HCC shares a similar mechanism of HCC pathogenesis with that of NAFLD. Acetaldehyde, an oxidation product of ethanol is a potent carcinogen driving the tumorigenesis by the formation of DNA adducts (106). Although the major pathway of metabolizing ethanol involves CYP2E1 in microsomes, acetaldehyde, and reactive oxygen species (ROS) are formed nevertheless (107). Interestingly, ethanol also induces steatosis by elevating the enzyme levels of *de novo* lipogenesis (DNL) and by suppressing the oxidation of fatty acid by downregulating PPARα (108, 109). In addition, progressive alterations in PNPLA3 and TM6SF2 genes, and micro RNA are known to promote steatosis, fibrosis, and cirrhosis in AFLD (110, 111). Thus similar to NAFLD-associated HCC, alcohol induces cirrhosis and promotes HCC development *via* the production of ROS, induction of chronic inflammation, activation of the immune response, leaky gut, and alteration of gene expression. However, the infiltration of inflammatory cells is found to be higher in AFLD (105, 112).

## Conclusion and Future Perspectives

HCC is a highly fatal cancer driven by multiple etiological factors, among which, fatty liver disease is emerging as a major cause worldwide. Based on the pathogenic origin, NAFLD has been strongly associated with glucose and lipid metabolism, whereas AFLD has been associated with a strong inflammatory response. NAFLD and AFLD share common molecular mechanisms in promoting HCC development, which involves vicious interplay between various pathways including immunological pathways, endocrine pathways, and metabolic pathways. However, there still exists a gap in the knowledge in understanding the molecular mechanisms of inflammation, genetic and epigenetic regulations, and genomic instability leading to hepatocarcinogenesis. Indeed, a comprehensive understanding of these diseases would aid in the identification of biomarkers and therapeutic targets leading to early detection and management.

Albeit, NAFLD- and AFLD-associated HCC are major challenging public health issues, it is preventable. The widely implemented curative approach is lifestyle alteration involving modifications in dietary habits and improving physical activity in case of NAFLD and alcohol abstinence in AFLD. Further personalized treatment strategies could improve healthcare and quality of patient care, thereby reducing the mortality rate. Alternatively, strategies like pharmacological treatment and bariatric surgery are also considered in patients unresponsive to lifestyle changes. Conclusively, it is important to develop diagnostic tests for the detection of early stages of HCC.

## Author Contributions

DS and DPK devised and wrote the manuscript. AS and DPK made the figures. All authors contributed to the article and approved the submitted version.

## Funding

This work was supported by Ramalingaswami Re-entry Fellowship to DPK from the Department of Biotechnology (DBT), Government of India.

## Conflict of Interest

The authors declare that the research was conducted in the absence of any commercial or financial relationships that could be construed as a potential conflict of interest.
